# RNase A Promotes Proliferation of Neuronal Progenitor Cells via an ERK-Dependent Pathway

**DOI:** 10.3389/fnmol.2018.00428

**Published:** 2018-11-26

**Authors:** Hsin-Yu Liu, Chiung-Ya Chen, Yun-Fen Hung, Hong-Ru Lin, Hsu-Wen Chao, Pu-Yun Shih, Chi-Ning Chuang, Wei-Ping Li, Tzyy-Nan Huang, Yi-Ping Hsueh

**Affiliations:** Institute of Molecular Biology, Academia Sinica, Taipei, Taiwan

**Keywords:** ERK activation, neural progenitor cells, neurogenesis, proliferation, RNase A

## Abstract

Members of the ribonuclease A (RNase A) superfamily regulate various physiological processes. RNase A, the best-studied member of the RNase A superfamily, is widely expressed in different tissues, including brains. We unexpectedly found that RNase A can trigger proliferation of neuronal progenitor cells (NPC) both *in vitro* and *in vivo*. RNase A treatment induced cell proliferation in dissociated neuronal cultures and increased cell mass in neurosphere cultures. BrdU (5-Bromo-2'-Deoxyuridine) labeling confirmed the effect of RNase A on cell proliferation. Those dividing cells were Nestin- and SOX2-positive, suggesting that RNase A triggers NPC proliferation. The proliferation inhibitor Ara-C completely suppressed the effect of RNase A on NPC counts, further supporting that RNase A increases NPC number mainly by promoting proliferation. Moreover, we found that RNase A treatment increased ERK phosphorylation and blockade of the ERK pathway inhibited the effect of RNase A on NPC proliferation. Intracerebroventricular injection of RNase A into mouse brain increased the population of 5-ethynyl-2'-deoxyuridine (EdU) or BrdU-labeled cells in the subventricular zone. Those RNase A-induced NPCs were able to migrate into other brain areas, including hippocampus, amygdala, cortex, striatum, and thalamus. In conclusion, our study shows that RNase A promotes proliferation of NPCs via an ERK-dependent pathway and further diversifies the physiological functions of the RNase A family.

## Introduction

The ribonuclease A (RNase A) superfamily consists of thirteen members in human (Cho et al., 2005; Gupta et al., 2013). Bovine RNase A, the best-characterized member of the RNase A family, was originally isolated from bovine pancreas. Human RNase 1, the RNase A ortholog, shares ~70% amino acid sequence identity with bovine RNase A (Beintema, 1998; Koczera et al., 2016). Hereafter in this report, RNase A describes either RNase A or RNase 1. The RNase A superfamily consists of secreted proteins that are expressed in a wide range of tissues, including brain (Gupta et al., 2013). They share similar primary sequences and a common disulfide-bonded tertiary conformation (Beintema, 1998). Though sequences are similar, the mammalian RNase A superfamily is one of the most rapidly-evolving gene families in mammals (Cho et al., 2005), which may be relevant to the versatile functions of this family, including anti-bacterial and anti-viral activities (Rosenberg, 2008; Gupta et al., 2013; Rademacher et al., 2016), inhibition or promotion of tumor growth and metastasis (Suzuki et al., 1999; Mironova et al., 2013, 2017; Chen et al., 2015; Attery et al., 2018; Wang et al., 2018), angiogenesis (Folkman and Klagsbrun, 1987; Gao and Xu, 2008; Sheng and Xu, 2016; Lyons et al., 2017), and cell proliferation (Li et al., 2013; Yu et al., 2017; Wang et al., 2018).

As a secreted protein, RNase A and other superfamily members are involved in the degradation of extracellular single-stranded RNA (ssRNA), though their enzymatic activities vary (Lyons et al., 2017). The extracellular ribonuclease activities of RNases have been suggested to play an anti-microbial function and to attenuate inflammation upon tissue injury (Rosenberg, 2008; Gupta et al., 2013). In addition to acting extracellularly, RNase A and other superfamily members are internalized via clathrin-dependent endocytosis and macropinocytosis (Chao and Raines, 2011). After internalization, RNases regulate multiple processes, including innate immune responses via endosomal Toll-like receptors and intracellular nucleotide receptors (Gupta et al., 2013; Liu et al., 2013), and induction of apoptosis via the caspase pathway (Chang et al., 2010). Studies also suggest that RNase 3 activates the caspase pathway by forming aggregates on the cell surface without internalization (Navarro et al., 2008, 2010). Thus, these studies indicate that RNase A superfamily members are involved in multiple cellular processes via both ribonuclease activity-dependent and -independent pathways.

Indeed, recent studies further suggested that a ribonuclease activity-independent pathway is critical for the RNase A superfamily to promote cell proliferation. Angiogenin, also known as RNase 5, has been shown to interact with several cellular proteins (Sheng and Xu, 2016; Lyons et al., 2017), including the transmembrane proteins heparan sulfate proteoglycan syndecan-4 (Skorupa et al., 2012), Plexin-B2 (Yu et al., 2017) and EGFR (Wang et al., 2018). The interaction between angiogenin and Plexin-B2 promotes proliferation of mouse embryonic teratocarcinoma P19 cells via activation of the AKT pathway (Yu et al., 2017). Like angiogenin, RNase 4 also promotes proliferation of P19 cells, though it is unclear whether Plexin-B2 is also involved in the action of RNase 4 (Li et al., 2013). In addition to P19 cells, angiogenin and RNase A also trigger cell proliferation of various pancreatic cancer cells via the EGFR-ERK pathway (Wang et al., 2018). Enzymatic dead mutants of RNase A and angiogenin still carry the ability to bind and activate EGFR (Wang et al., 2018). Thus, the abilities of RNase A and angiogenin to promote cell proliferation could be independent of its enzymatic activity. Moreover, promoting cell proliferation is one of common functions of RNase A, angiogenin and RNase 4.

To demonstrate the involvement of endogenous ssRNA in stimulating neuronal Toll-like receptor 7 (TLR7), we previously added RNase A to digest ssRNA in cortical and hippocampal mixed cultures. We found that RNase A treatment neutralizes TLR7-mediated dendritic withdrawal of cultured neurons, because the dendritic length of wild-type neurons, but not *Tlr7*^−/−^ neurons, was increased in the presence of RNase A (Liu et al., 2013). In addition, we noticed that cell density was increased in RNase A-treated cultures. We hypothesized that RNase A treatment promotes cell proliferation of neuronal cultures. To explore this possibility, here, we investigated whether numbers of proliferating cells are increased in response to RNase A treatment and which types of cells are expanded *in vitro* in the presence of RNase A. Our data suggest that neuronal progenitor cells (NPCs) respond to RNase A. We also examined the involvement of ERK in RNase A downstream signaling. Finally, we demonstrate the effect of RNase A on promoting EdU incorporation *in vivo*. Our study suggests an unexpected activity of RNase A in triggering NPC proliferation.

## Materials and methods

### Chemicals

RNase A was purchased from Invitrogen (12091-021) and Qiagen (19101). Bovine serum albumin (BSA, A4919), bromodeoxyuridine (BrdU, B5002), cytosine arabinoside (Ara-C, C1768), U0126, and poly-L-lysine (P2636, 30,000-70,000 molecular weight) were purchased from Sigma-Aldrich. EdU (A10044) was purchased from Invitrogen.

### Antibodies

Primary antibodies used in this report are summarized in Table S1. Alexa Fluor 488- and Alexa Fluor 594-conjugated secondary antibodies were purchased from Invitrogen.

### Animals

Wild type C57BL/6 mice were housed in the animal facility of the Institute of Molecular Biology, Academia Sinica, under pathogen-free conditions and a 14 h light/10 h dark cycle with controlled temperature and humidity. Mixed gender embryos were used for neuronal cultures. Adult male mice were used for *in vivo* analysis. All animal experiments were performed with the approval (Protocol Number 13-02-520) of the Academia Sinica Institutional Animal Care and Utilization Committee and in strict accordance with its guidelines and those of the Council of Agriculture Guidebook for the Care and Use of Laboratory Animals.

### Primary culture

Our neuronal cultures were prepared as described previously (Hung et al., 2018). Briefly, cerebral cortices and hippocampi of mouse embryos at E17-18 were dissected, dissociated with trypsin and cultured in Neurobasal/DMEM (1:1) with 2% B27 supplement, 0.5 mM glutamine, and penicillin-streptomycin. EGF and FGF were not included in the medium. RNase A was added at 1 DIV. After treatment for 3 days, cultures were harvested for analysis. Our culture medium contains a final concentration of 2,500 μg/ml bovine serum albumin (BSA, A4919, Sigma) (Chen et al., 2008). An additional 100 μg/ml BSA (A4919, Sigma) was added as a negative control for RNase A treatment in this report. We controlled cell density at 2 × 10^5^ cells/well in 12-well plates with 18-mm coverslips precoated with 1 mg/ml poly-L-lysine (P2636, 30,000-70,000 molecular weight, Sigma-Aldrich). These culture conditions enrich the neuronal population. The percentage of glial cells (GFAP^+^ cells) in our cultures is around 2–3% (Liu et al., 2013). To inhibit NPC proliferation, 1 μM Ara-C was added to the cultured neurons after 24 h RNase A treatment.

### Time-lapse imaging

Neurons were cultured with a density of 1.5 × 10^5^ cells/cm^2^ on 36-mm coverslips. Four hours after seeding, neurons were transferred to POC-R cell cultivation system (LaCon) for time-lapse recording. Before recording, neurons were inoculated in LSM510META-NLO system at 37°C with 5% CO_2_ supplement for at least 2 h for system balance. The recording was carried out using Plan-Apochromat 20x/NA0.8 M27 objective lens (Carl Zeiss, Inc.) with 0.5% laser energy in a live-cell incubation chamber. Images were acquired every 3 min for 96 h with a resolution of 1,024 × 1,024 pixels. Results were then processed for publication using ImageJ (NIH) with minimal adjustment of brightness or contrast applied to the whole images.

### Neurosphere culture

NPCs derived from a mixture of mouse cerebral cortices and hippocampi of E17-18 embryos were cultured at a density of 8 × 10^4^ cells/well in flat-bottomed 96-well plates and maintained in F12/DMEM (1:1) with 2% B27 supplement. EGF and FGF were not included in the medium. RNase A or BSA was added to the medium and incubated for 9 days. The cultures were visualized with an Image Xpress Micro system (Molecular Devices) equipped with a 10x objective lens (Plan Fluor; Nikon). The number of neurospheres per well and the area of each neurosphere were measured using software provided by the Image Xpress Micro system. To investigate cell growth of neurospheres in response to different dosages of RNase A, the averaged area of each neurosphere was analyzed for each well. In addition, all neurospheres were divided into nine groups based on their size, ranging from 0-10,000 to >80,000 μm^2^, using Microsoft Excel software (COUNTIFS function), and the percentage of each group was calculated. Experiments were independently repeated more than three times.

### ERK phosphorylation

At 1 DIV, dissociated neuronal cultures were treated with RNase A (100 μg/ml) for 0, 10, 20, 30, and 60 min. To investigate the dosage effect of U0126, cultures were treated with U0126 at different dosages (0, 5, or 10 μM) for 30 min followed by treatment with or without RNase A (100 μg/ml) for 20 min. For neurosphere cultures, 4 DIV cells were pretreated with U0126 (10 μM) or DMSO control for 30 min, followed by RNase A (100 μg/ml) stimulation for 0, 10, 20, 30, and 60 min. Total cell lysates were harvested and subjected to immunoblot analysis using phospho-ERK, pan ERK, and VCP antibodies.

### *in vitro* labeling of BrdU and immunofluorescence staining

BrdU was added to cultured neurons at a concentration of 10 μM for 2 h before fixation with 4% paraformaldehyde (PFA) and 4% sucrose in phosphate buffered saline (PBS) for 15 min at room temperature. After washing with PBS, cells were denatured with 2N HCl for 30 min at 37°C. Cells were then washed twice with PBS and permeabilized with 0.1% Triton-X 100 (Sigma-Aldrich) in PBS for 10 min at room temperature and blocked with 5% BSA (A7906; Sigma-Aldrich) in PBS for 30 min. Fixed cells were incubated with primary antibodies diluted in 5% BSA/PBS overnight at 4°C. After washing with PBS, neurons were incubated with Alexa Fluor-488- and 594-conjugated secondary antibodies for 2 h at room temperature. Samples were then mounted with Vectashield mounting medium (H-1000; Vector Laboratories) and visualized with an Axio Imager-Z1 or M2 microscope (Carl Zeiss). To quantify the effect, five images were captured randomly and blindly from each coverslip under a 10x objective lens/NA 0.45 or 20x objective lens/NA 0.8 (Plan Apochromat; Carl Zeiss). The average of five images represented the result of each coverslip. For the Z1 microscope, immunofluorescence images were captured with a digital AxioCam MRm camera driven by the digital image processing software AxioVision.

### Stereotactic brain surgery and intracerebroventricular injection

Three-month-old male C57BL/6 mice were deeply anesthetized with zoletil (0.25 mg/kg bodyweight) and xylazine (0.08 mg/kg bodyweight) in 0.9% NaCl. Mice were stereotactically implanted with a stainless steel guide cannula (outer diameter 0.55 mm/inner diameter 0.31 mm/length 8 mm) into the right lateral cerebroventricle (0.54 mm posterior to the bregma, 1.2 mm lateral to midline and 2.21 mm below the skull). Three screws fixed the guide tube to the skull and dental cement was used to encase the screws and guide tube. After two weeks of recovery, BSA (90 μg/μl) or RNase A (90 μg/μl; QIAGEN) was dissolved in artificial cerebrospinal fluid (aCSF) with 0.01% Fast Green and injected into the lateral cerebroventricle at a volume of 2 μl for 10 min (rate 0.2 μl/min) under anesthesia with 2.5% isoflurane. The injector was removed 10 min later.

### EdU *in vivo* labeling and quantification

After intracerebroventricular injection of BSA or RNase A (1–4 times), each group of mice received a single intraperitoneal injection of EdU (stock: 10 mg/ml in 10% DMSO) at a dosage of 100 mg/kg bodyweight to monitor cell proliferation. Mouse brains were harvested 8 days after injection of BSA or RNase A. After perfusion with PBS and 4% PFA/PBS, brains were dissected and postfixed with 4% PFA/PBS overnight at 4°C. Fifty-μm-thick coronal sections were sliced with a LEICA VT 1200S microtome. EdU staining was conducted using the Click-iT^TM^ Plus EdU Imaging kit with Alexa Fluor 488 (C10637; Invitrogen) according to the manufacturer's instructions. Brain sections were permeabilized with 0.5% Triton X-100 in PBS for 30 min. After washing twice with 3% BSA in PBS, brain sections were incubated with a Click-iT^TM^ reaction cocktail for 30 min while protected from light. The sections were washed twice with 3% BSA in PBS and mounted with DAPI in Vectashield mounting medium (H-1000; Vector Laboratories). Samples were visualized with an Axio Imager M2 microscope (Carl Zeiss) equipped with a 10x objective lens/NA 0.45 (Plan Apochromat; Carl Zeiss). Fluorescence images were captured with a digital Rolera EM-C^2^ camera driven by the digital image processing software Zen Blue. All images were converted into 8-bit (Figures S2A, S3A) and adjusted with a threshold (“Moments” and “B&W” modes in “Dark background”) (Figures S2B, S3B) using ImageJ software. The area of fluorescence reactivity was analyzed using “Analyze Particles” with the setting of 30-infinity pixel units and 0.01–1.00 circularity (Figures S2C, S2D, S3C–E). The quantitative results were manually corrected to remove artifacts, such as high background or false signals (Figures S2F, S3F). The data were then transferred to Excel to calculate the ratio of number of EdU puncta to total area of brain at the lateral ventricle (Figures S2E,F) or both sides of the hippocampus (Figure S3B), as well as the percentage area of EdU signal. Examples of the quantification process are available in Figures S2, S3.

### *in vivo* labeling of BrdU and immunofluorescence staining

After intracerebroventricular injection of BSA or RNase A, each group of mice received intraperitoneal injection of BrdU (stock: 10 mg/ml in 10% DMSO) at a dosage of 50 mg/kg bodyweight, as indicated in Figure 9A. Mouse brains were harvested 30 days after injection of BSA or RNase A. After perfusion with PBS and 4% PFA/PBS, brains were dissected and postfixed with 4% PFA/PBS overnight at 4°C. For BrdU staining, sections were denatured with 2N HCl for 30 min at 37°C. After washing twice with PBS, brain sections were permeabilized with 0.5% Triton-X 100 (Sigma-Aldrich) in PBS for 10 min at room temperature and blocked with blocking solution (1% BSA, 3% horse serum, and 0.3% Triton-X 100 in PBS) for 30 min. The sections were then incubated with the anti-BrdU antibody (ab6326, Abcam) and other primary antibodies diluted in blocking solution overnight at 4°C. After washing with PBS, the sections were incubated with Alexa Fluor-488- and 594-conjugated secondary antibodies (1:500) for 2 h at room temperature. The sections were then washed twice with PBS and mounted with DAPI in Vectashield mounting medium (H-1000; Vector Laboratories). Samples were visualized with an Axio Imager M2 microscope (Carl Zeiss) equipped with a 10x objective lens/NA 0.45 (Plan Apochromat; Carl Zeiss) and a confocal microscope (LSM 700; Carl Zeiss) equipped with a 20x/NA 0.8 (Plan-Apochromat; Carl Zeiss) objective lens.

### Statistical analysis

For culture, each set of experiment was independently repeated 3–4 times using different litters. For each experiment, five fluorescence images were captured randomly and blindly from each coverslip. The average of the data of five images represented the result of each coverslip. All graphs were plotted and analyzed using GraphPad Prism 5.0 or 7.0. Data are presented as the mean plus SD. *P* values of < 0.05 were considered significant. In Figures 7A,B, the experiment was repeated three times using independent neuronal culture. The band intensity of p-ERK and ERK in each sample was quantitated using imageJ. After obtained p-ERK/ERK ratio in each sample, all the p-ERK/ERK ratios were further normalized to the control. For multiple group comparisons, we applied the one-way analysis of variance (ANOVA) or two-way ANOVA with Bonferroni's test. For two group comparisons, the normality of each group was analyzed using a Shapiro-Wilk normality test. Statistical analysis of the normally distributed groups was performed using unpaired *t*-tests. Statistical analysis of non-normally distributed groups was performed using two-tailed nonparametric tests (Mann-Whitney tests). The variance between groups was not statistically compared. Data collection and analysis were conducted randomly and blind through relabeling of the samples by other lab members.

## Results

### RNase a increases cell number of NPCs in dissociated neuronal cultures

**Figure 1:**
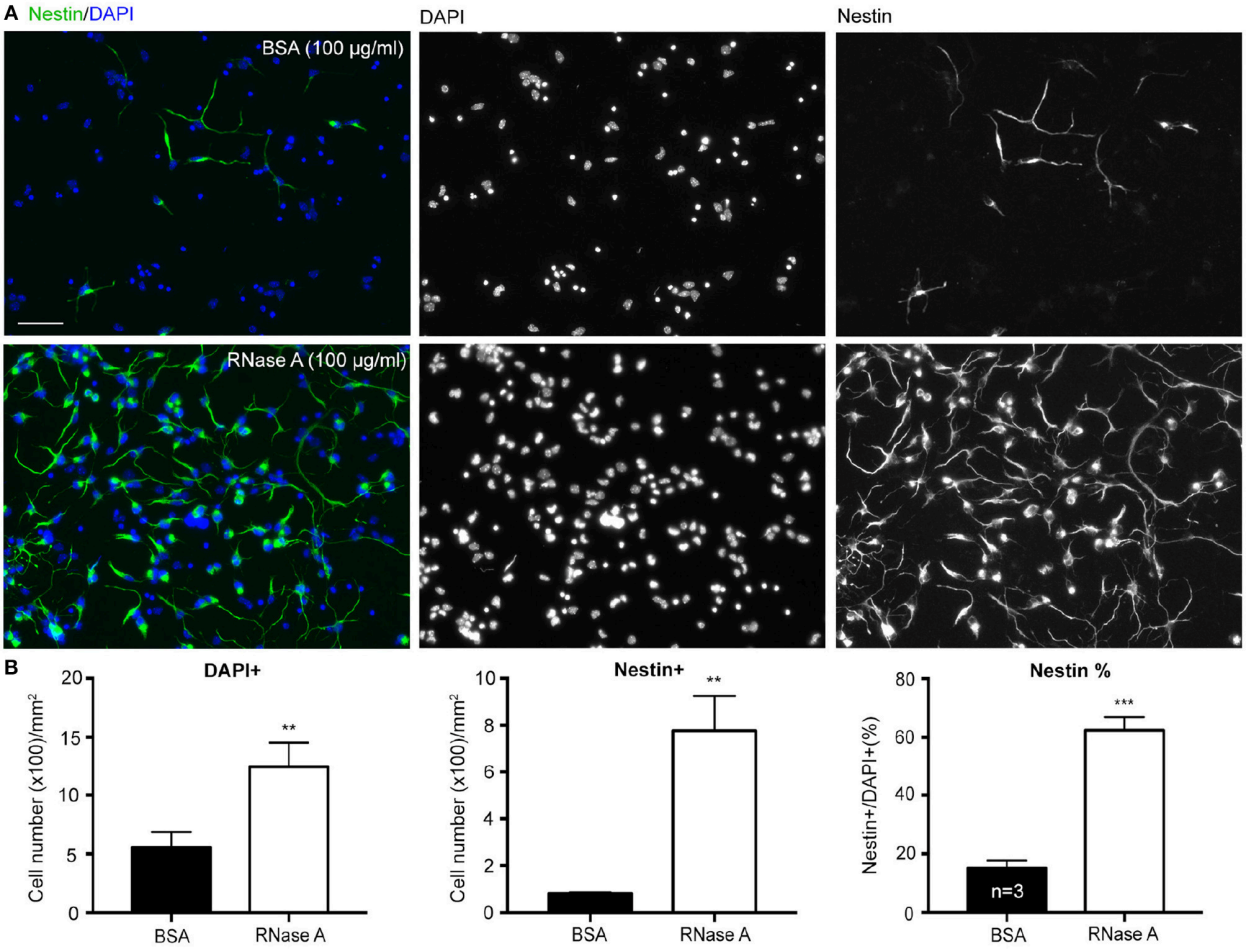
RNase A treatment increases numbers of NPCs in dissociated neuronal cultures. Mixed mouse cortex and hippocampus cultures were treated with 100 μg/ml BSA or Invitrogen RNase A at 1 DIV and grown for 3 days. **(A)** Representative images of immunostaining with Nestin, an NPC marker, are shown. Counter-staining with DAPI was performed to label cell nuclei. The number of DAPI^+^ cells represents the total cell number. **(B)** Quantifications, including the number of total DAPI^+^ cells, the number of Nestin^+^ cells and the percentage of Nestin^+^ cells in total DAPI^+^ cells. Mean and SD of three independent experiments are shown. Scale bars, 50 μm. Statistical analyses were performed using unpaired *t*-tests. ^**^P < 0.01; ^***^P < 0.001.

To confirm the role of ssRNA in regulation of neuronal morphology, RNase A was added to cortical and hippocampal mixed neuronal cultures to digest ssRNA in cultures (Liu et al., 2013). In addition to promoting dendritic outgrowth (Liu et al., 2013), we noticed when RNase A (final concentration 100 μg/ml) purchased from Invitrogen was added to cortical and hippocampal mixed neuronal cultures at 1 day *in vitro* (DIV), the cell density indicated by DAPI counter-staining was increased at 4 DIV (Figures 1A,B). Our cultures were dissociated neuronal cultures grown in Neurobasal/DMEM medium with B27 supplement (Hung et al., 2018). BSA (final 2,500 μg/ml) was the major protein component in the medium (Chen et al., 2008). Thus, we first used an additional 100 μg/ml of BSA as a negative control for RNase A treatment. Compared with BSA control, RNase A increased total cell number by more than 200% (Figure 1B). Immunostaining using Nestin antibody showed that the number of Nestin^+^ cells was noticeably increased in RNase A-treated groups (Figures 1A,B), suggesting an increase of NPC number upon RNase A treatment. The increased number of NPCs was roughly equal to the increased total cell number (Figure 1B, left vs. middle). When we calculated the percentage of Nestin^+^ cells, we also found that the population of Nestin^+^ cells increased from ~20% in BSA groups to 60% in RNase A-treated groups (Figure 1B, right). Thus, compared with BSA control, RNase A treatment increased the population of Nestin^+^ NPCs in dissociated neuronal cultures.

**Figure 2:**
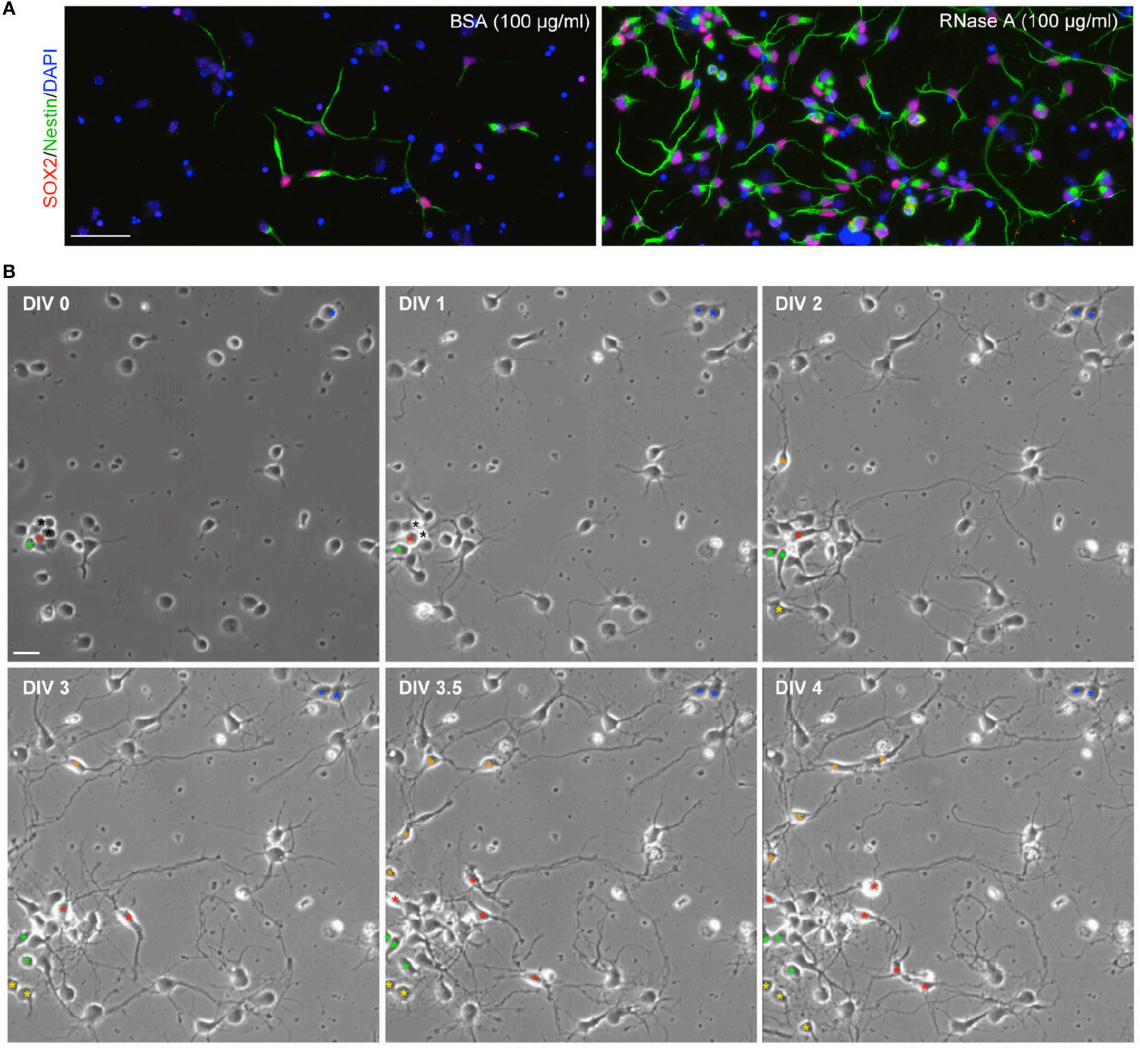
Dividing NPCs are present in neuronal cultures. **(A)** SOX2^+^Nestin^+^ cells are present in neuronal cultures. RNase A-induced Nestin-positive cells are also SOX2-positive. **(B)** Live recording of neuronal culture from DIV 0 to 4. The video is available as Movie S1. Bright-field images at the indicated time-points are shown. Asterisks indicate NPCs or their daughter cells. Asterisks of the same color indicate the same lineage of cells. Black asterisks at DIV 0 and 1 indicate two cells, which were dead at DIV1. Scale bars, **(A)** 50 μm; **(B)** 20 μm.

Since our culture medium was originally designed for neuronal culture (see Materials and Methods), we did not expect to find Nestin^+^ NPCs under our culture conditions. To confirm that the observed cells were NPCs, we also performed immunostaining with SOX2 antibody, another NPC marker. Indeed, Nestin and SOX2 co-existed in the same cells (Figure 2A), supporting that the proliferated cells are NPCs. If NPCs are indeed present in our cultures, we would expect to see dividing cells in our cultures. To assess this possibility, we conducted time-lapse recording from DIV 0 to 4. We found that dividing cells were easily visible in our neuronal cultures. We present an example containing three NPCs in a single field in Figure 2B and Movie S1. One of the NPCs underwent a single division and differentiated into neurons (Figure 2B, blue asterisk). Two NPCs underwent several divisions and produced multiple daughter cells (Figure 2B,red and green asterisks). These NPCs typically had two or three long thick processes and exhibited highly motile nuclei moving along their processes (Movie S1). The morphology of these dividing cells is similar to the Nestin^+^ cells revealed by immunostaining (Figure 1). These data suggest that Nestin- and SOX2-positive dividing NPCs are present in our neuronal cultures.

**Figure 3:**
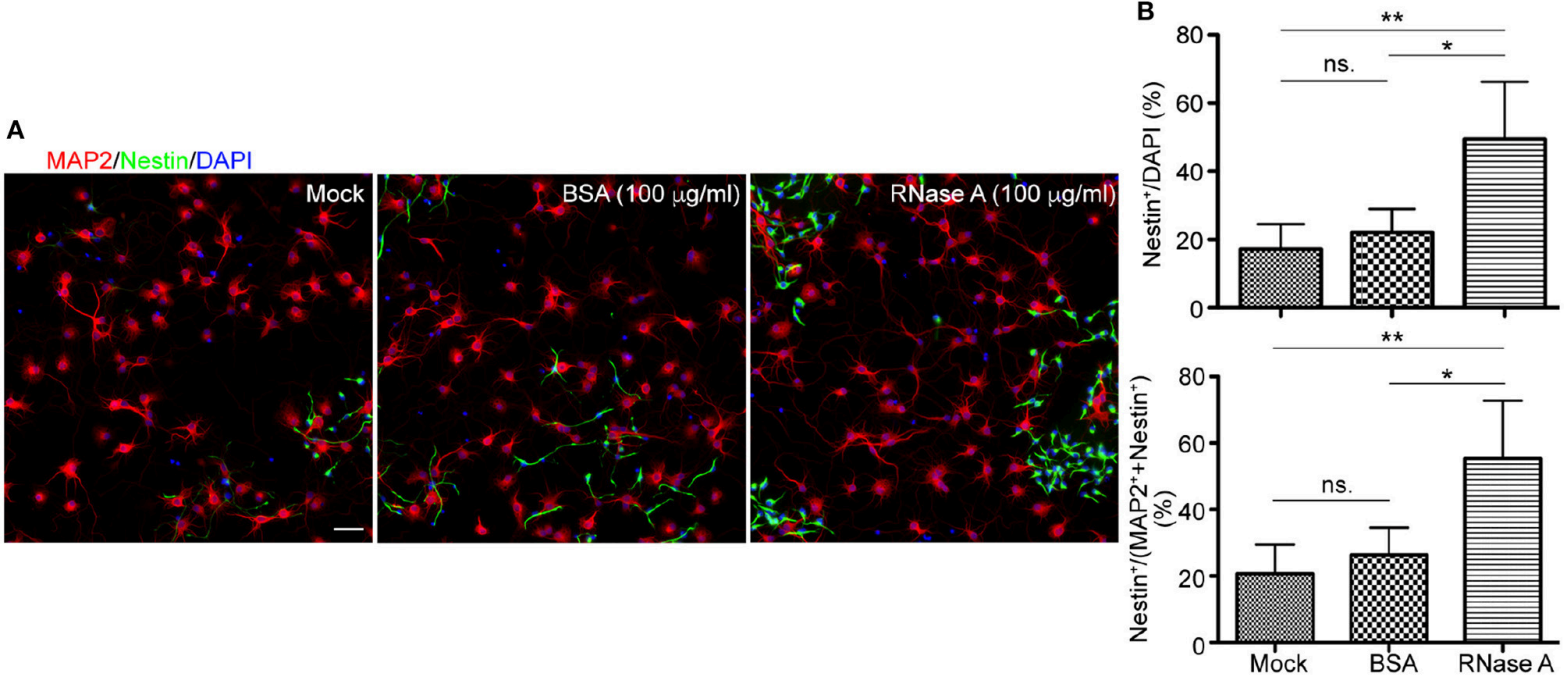
Qiagen RNase A also increases the NPC population in neuronal cultures. Qiagen RNase A (100 μg/ml) and BSA (100 μg/ml) were added into neuronal cultures at 1 DIV for 3 days. Mock control without adding any protein was also included. At 4 DIV, cells were fixed and immunostained with Nestin and MAP2 antibodies. Counter-staining with DAPI was performed to determine the total cell number. **(A)** Representative images. Scale bars, 50 μm. **(B)** Quantifications of the percentage of Nestin^+^ cells in the total DAPI^+^ cells (upper) and the sum of MAP2^+^ and Nestin^+^ cells (bottom). Mean and SD of four experiments are shown. Statistical analyses were performed using one-way ANOVA. *P < 0.05; **P < 0.01; ns., not significant.

To further confirm the effect of RNase A on NPC proliferation, we tested RNase A purchased from different company, namely Qiagen. Similar to RNase A from Invitrogen (Figure 1), Qiagen RNase A also increased NPC population in neuronal cultures, as the percentage of Nestin^+^ NPCs in total cells was increased by RNase A compared to BSA control (Figures 3A,B upper). Thus, both Invitrogen and Qiagen RNase A increased NPC number in cultures compared with BSA control.

In addition to BSA control, mock control without adding extra protein was also included (Figure 3). It was clear that the population of Nestin^+^ cells in BSA treated group was comparable to mock control and that RNase A treatment increased the percentage of Nestin^+^ cells compared with mock control (Figure 3B, upper). Thus, no matter comparing with mock control or BSA control, RNase A treatment noticeably increases the population of Nestin^+^ cells in dissociated neuronal cultures.

We also performed a double staining using antibodies against Nestin and MAP2, a neuronal marker (Figure 3). Neurons and NPCs are the two major populations in our cultures. Compared with BSA and mock controls, we found that the population of Nestin^+^ cells was increased from ~20% to ~40–70% of total Nestin^+^ and MAP2^+^ cells (Figure 3B). Thus, the population of Nestin^+^ cells was increased upon RNase A treatment no matter we used DAPI staining to count the total cell number or the sum of Nestin^+^ and MAP2^+^ cells to represent the major populations in cultures.

### RNase a promotes NPC proliferation

**Figure 4:**
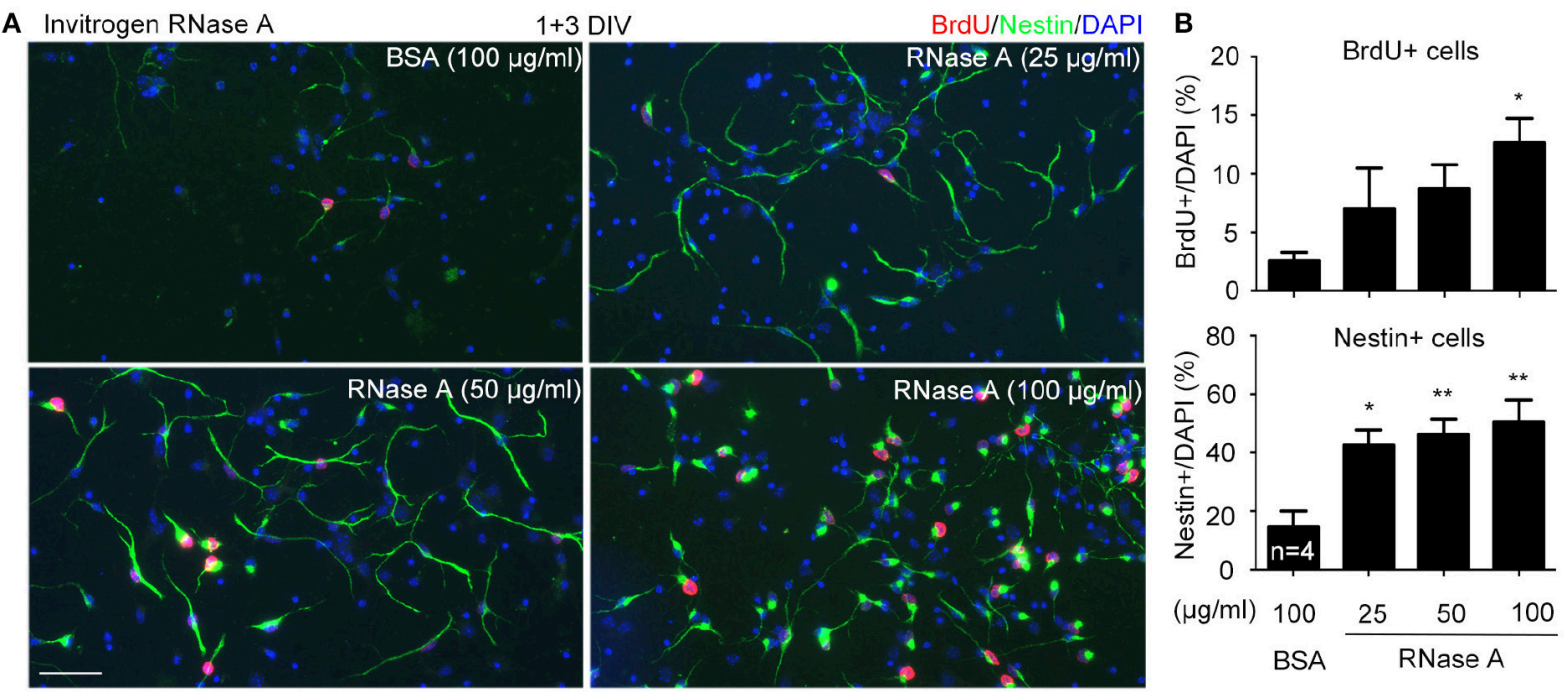
Dosage effect of RNase A on NPC proliferation. Different amounts (25, 50, 100 μg/ml) of Invitrogen RNase A were added to mouse cortex and hippocampus neuronal cultures at 1 DIV and grown for 3 days. BSA (100 μg/ml) was included as a control. BrdU was added to cultures 2 h before harvesting. Immunostaining was performed with BrdU and Nestin antibodies. Counter-staining with DAPI was performed to determine the total cell number. **(A)** Representative images. **(B)** Quantifications of the percentage of BrdU^+^ cells (upper) and Nestin^+^ cells (bottom) in total cell number. Data represent mean plus SD. The experiments were independently repeated four times. Scale bar, 50 μm. Statistical analyses were performed using one-way ANOVA. *P < 0.05; **P < 0.01.

Increased NPC population in our cultures suggests that RNase A treatment likely promotes proliferation. To test the effect of RNase A on NPC proliferation, we added 5-bromo-2′-deoxyuridine (BrdU, a thymidine nucleoside analog) into cultures 2 h before harvesting. Meanwhile, the dosage effect of RNase A was also investigated. We found that similar to the effect on Nestin^+^ cells, RNase A treatment indeed increased the population of BrdU^+^ cells (Figures 4A,B). Twenty five μg/ml RNase A was sufficient to increase the population of Nestin^+^ cells (Figures 4A,B). It also increased BrdU labeling in the cultures, though the difference was not yet significant. These data suggest that RNase A treatment promotes BrdU incorporation by NPCs.

**Figure 5:**
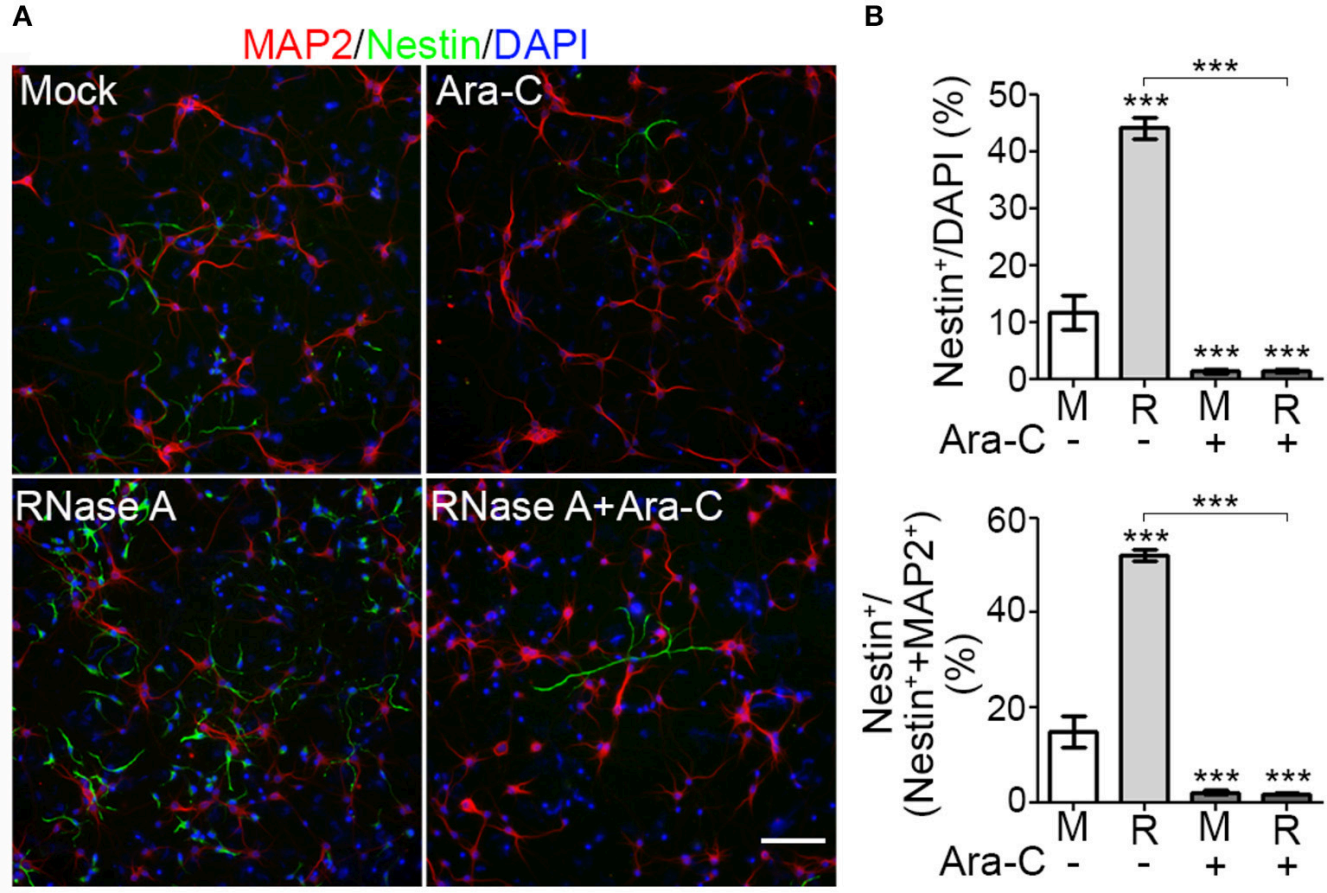
Proliferation inhibitor Ara-C blocks the effect of RNase A on NPC proliferation. Mixed mouse cortex and hippocampus cultures were treated with 100 μg/ml Qiagen RNase A (R) at 1 DIV. Mock control (M) represents samples to which no extra material had been added. At 2 DIV, Ara-C (final 1 μM) was added into the culture. After two more days, cultures were harvested and immunostained using MAP2 and Nestin antibodies. DAPI staining was also performed to label cell nuclei. (**A**) Representative images. (**B**) Quantification of the percentage of Nestin^+^ NPCs in total cells (indicated by DAPI stain, upper panel) and in the sum of MAP2^+^ neurons and Nestin^+^ NPCs (lower panel). Five non-overlapping images under the microscope were randomly selected to determine the averages of cell numbers. Means and SD of three experiments are shown. Scale bars, 100 μm. Statistical analyses were performed using two-way ANOVA with Bonferroni's test. ***P < 0.001.

To reinforce the evidence for an effect of RNase A on NPC proliferation, we added the proliferation inhibitor cytosine arabinoside (Ara-C) to our cultures. Compared with mock control, RNase A treatment increased the percentage of Nestin^+^ cells from < 20% to ~44% of total cells (indicated by DAPI staining) and ~52% of the sum of MAP2^+^ neurons and Nestin^+^ NPCs (Figures 5A,B). Addition of 1 μM Ara-C efficiently reduced the numbers of Nestin^+^ cells in both mock control and RNase A-treated cultures (Figure 5B). Whenever we added Ara-C to cultures, the percentage of Nestin^+^ NPCs was reduced to ~2% that of total cells or the sum of MAP2^+^ neurons and Nestin^+^ NPCs (Figure 5B). Taken together, the results of our BrdU incorporation assay and Ara-C inhibition experiment indicate that RNase A treatment promotes NPC proliferation.

### RNase a also increases neurosphere cell mass

**Figure 6:**
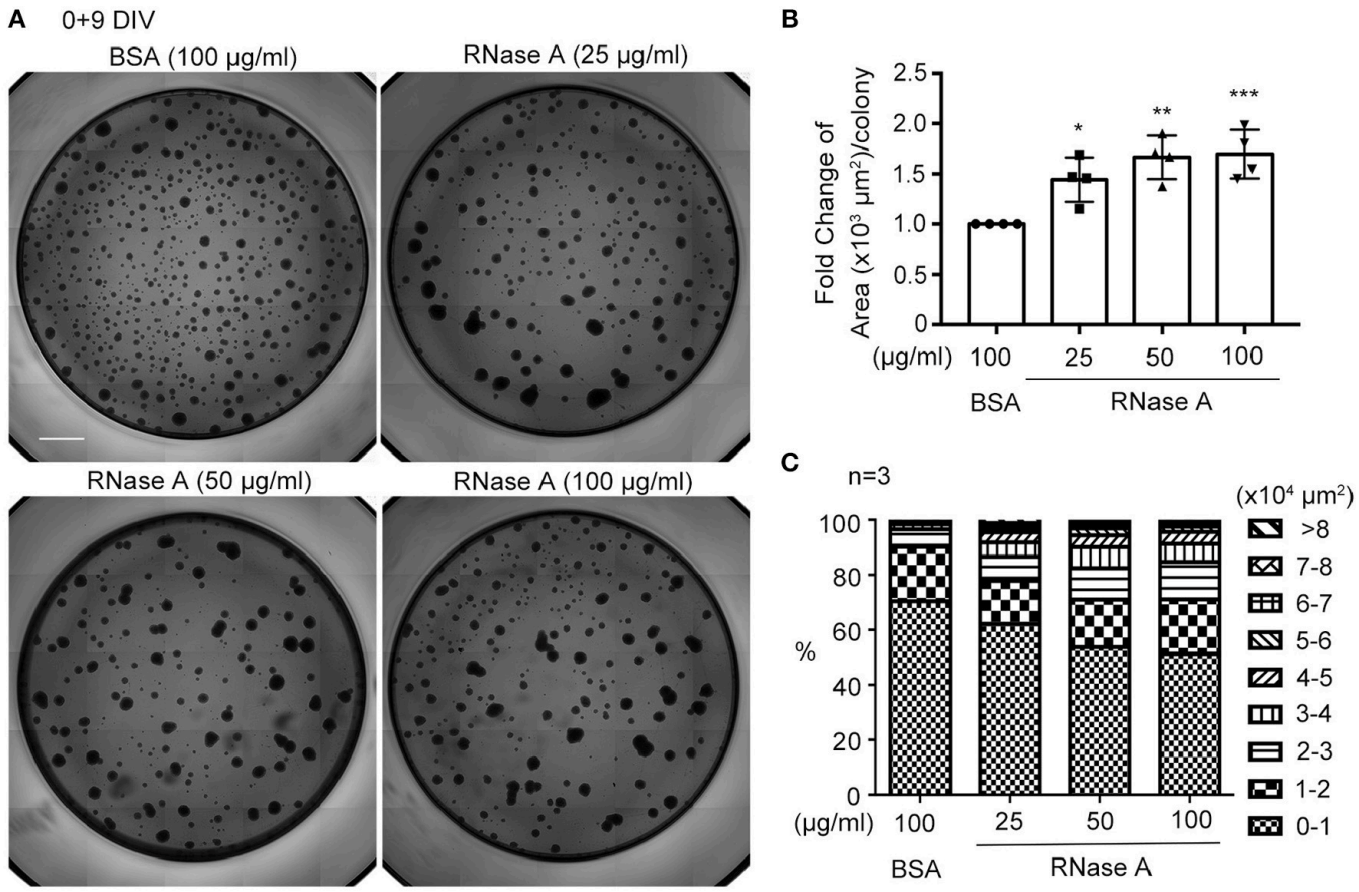
RNase A treatment promotes the growth of neurospheres. **(A)** Photographs of primary neurospheres treated with RNase A (Invitrogen, 25, 50, and 100 μg/ml) and grown for 9 days in 96-well plates. The medium did not contain the typical growth factors, such as EGF and FGF2, for NPCs. Scale bar, 300 μm. **(B)** Quantification of averaged area of each neurosphere colony in the photographs. The experiments were independently repeated four times. Mean and SD are shown. Statistical analyses were performed using one-way ANOVA. *P < 0.05; **P < 0.01; ***P < 0.001. **(C)** The effect of RNase A on the size of neurospheres. Neurospheres were divided into 9 groups based on their size from 0-10,000 to >80,000 μm^2^, and the percentages of each group under different dosages of RNase A treatment are shown. The experiments were independently repeated three times. Means of the populations are shown.

We utilized dissociated neuronal cultures for all of the previously described analyses. We then examined the effect of RNase A on neurosphere cultures. Different amounts of RNase A (25, 50, or 100 μg/ml) were added to neurosphere cultures in the absence of EGF and FGF2 (Figures 6A–C). Nine days after culture, the averaged area of each neurosphere had increased in a dosage-dependent manner in the 25–100 μg/ml RNase A-treated groups (Figure 6B). We also quantified the populations of differently sized neurospheres and found that the numbers of larger colonies had increased in the presence of RNase A (Figure 6C). Thus, RNase A also promotes the growth of neurospheres.

### ERK activation is involved in rnase a-mediated neurogenesis

**Figure 7:**
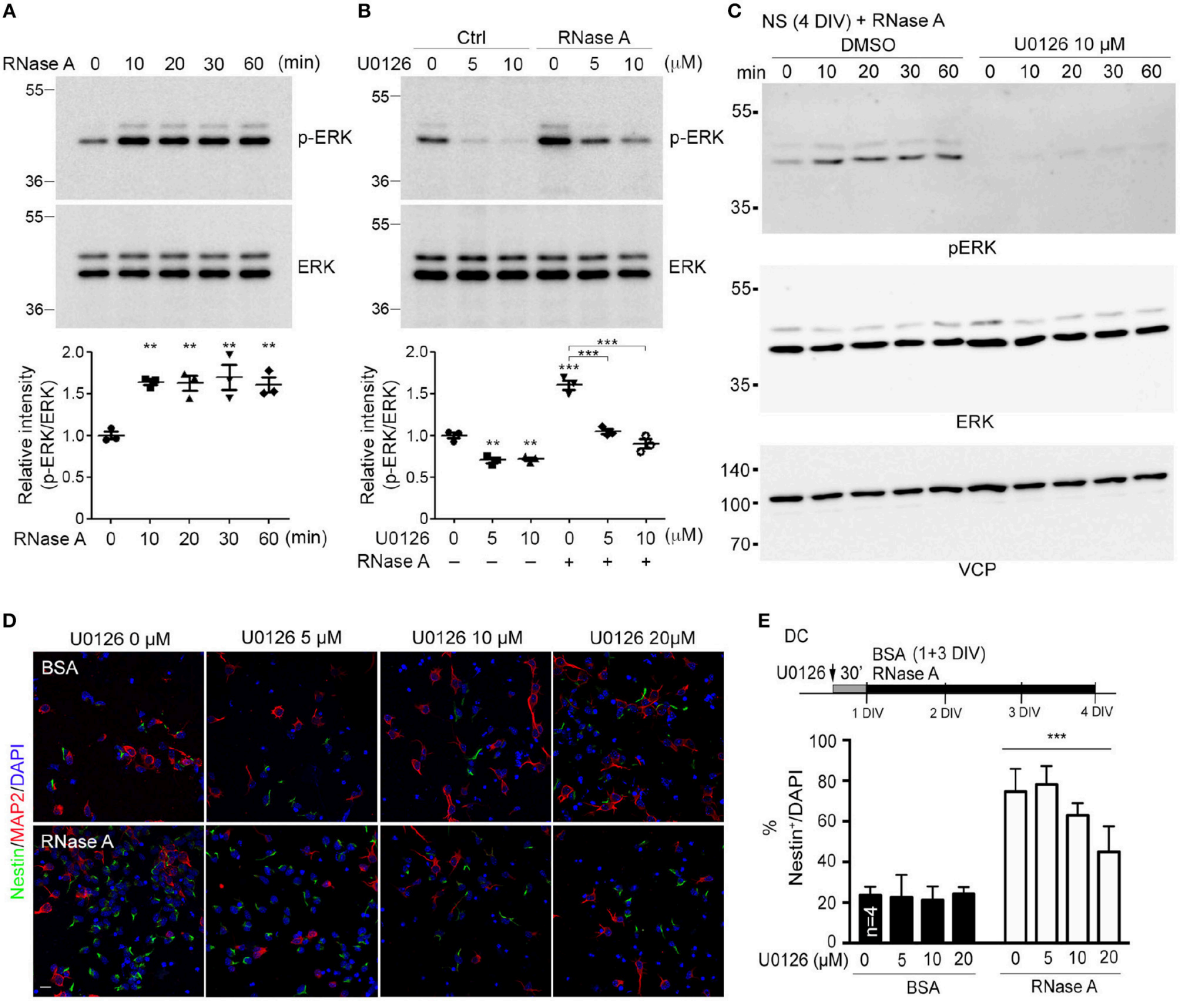
RNase A induces NPC proliferation through the ERK pathway. **(A)** At 1 DIV, dissociated cortical and hippocampal cultures were treated with 100 μg/ml RNase A (Qiagen) and harvested at different time-points, as indicated. ERK activities were detected by means of immunoblotting with antibody recognizing phosphorylated ERK1/2 (pERK). **(B)** Pretreatment with U0126 (a MEK1/2 inhibitor) at dosages of 0, 5, or 10 μM for 30 min was performed to examine the specificity of RNase A for ERK activation. RNase A or BSA control (100 μg/ml) was added 20 min before harvesting. Quantification data shown at the bottoms of **(A)** and **(B)** are mean and SEM of three independent experiments. Statistical analyses were performed using one-way ANOVA **(A)** and two-way ANOVA **(B)**. **P < 0.01; ***P < 0.001. **(C)** ERK activation by RNase A in neurospheres (NS). NS were pretreated with U0126 (10 μM) for 30 min, followed by RNase A, and harvested at the indicated time-points (0, 10, 20, 30, and 60 min). **(D)** U0126 blocks the NPC proliferation induced by RNase A in a dosage-dependent manner. Cultures were dual-stained with Nestin (NPCs, green) and MAP2 (neurons, red). Scale bar, 20 μm. **(E)** Quantification of **(D)**. The experiments were independently repeated four times. Mean and SD are shown. Statistical analyses were performed using two-way ANOVA. *** *P* < 0.001 in **(E)** for comparison of Nestin^+^ cells between RNase A-treated alone and RNase A plus U0126 double-treatment groups.

A very recent study showed that RNase A and angiogenin activate the EGFR-ERK pathway and AKT to promote proliferation of pancreatic cancer cells (Wang et al., 2018). We wondered whether RNase A also activates these pathways in NPCs. To investigate this possibility, we monitored ERK activity (indicated by ERK phosphorylation levels) upon RNase A treatment. In dissociated cortical and hippocampal neurons, phospho-ERK signals were increased 10 min after RNase A treatment and lasted for at least 60 min (Figure 7A). To confirm that the increased ERK phosphorylation was specifically caused by activation of the ERK pathway, we added U0126 (a MEK1/2 inhibitor) to the culture 30 min before RNase A treatment. Indeed, U0126 effectively eliminated RNase A-induced ERK phosphorylation in dissociated cortical and hippocampal neurons (Figure 7B). We also investigated the involvement of ERK in the response of neurospheres to RNase A. As for dissociated cultures, RNase A treatment increased ERK activity in neurospheres (Figure 7C, left panel), and U0126 treatment blocked the ERK activity induced in them by RNase A (Figure 7C, right panel). We also examined the activity of AKT upon RNase A treatment. In contrast to ERK, AKT activation upon RNase A stimulation was not obvious (Figure S1). Thus, our evidence suggests that RNase A treatment activates the ERK pathway but does not promote AKT activity.

To further investigate the role of ERK in RNase A-dependent neurogenesis, we monitored the effect of U0126 on the number of Nestin^+^ cells upon RNase A treatment. Different doses of U0126 (0, 2, 5, 10, or 20 μM) were added to dissociated cultures 30 min before adding RNase A or BSA at 1 DIV. Three days later, the cultures were immunostained with Nestin and MAP2 antibodies and counter-stained with DAPI to determine their populations of Nestin^+^ cells. Consistent with its effect of reducing ERK phosphorylation, U0126 exhibited the dosage-dependent effect of decreasing the numbers of Nestin^+^ cells induced by RNase A (Figures 7D,E). These results suggest that RNase A treatment induces ERK activation and promotes NPC proliferation in cultures.

### RNase a promotes NPC proliferation in mouse brains

**Figure 8:**
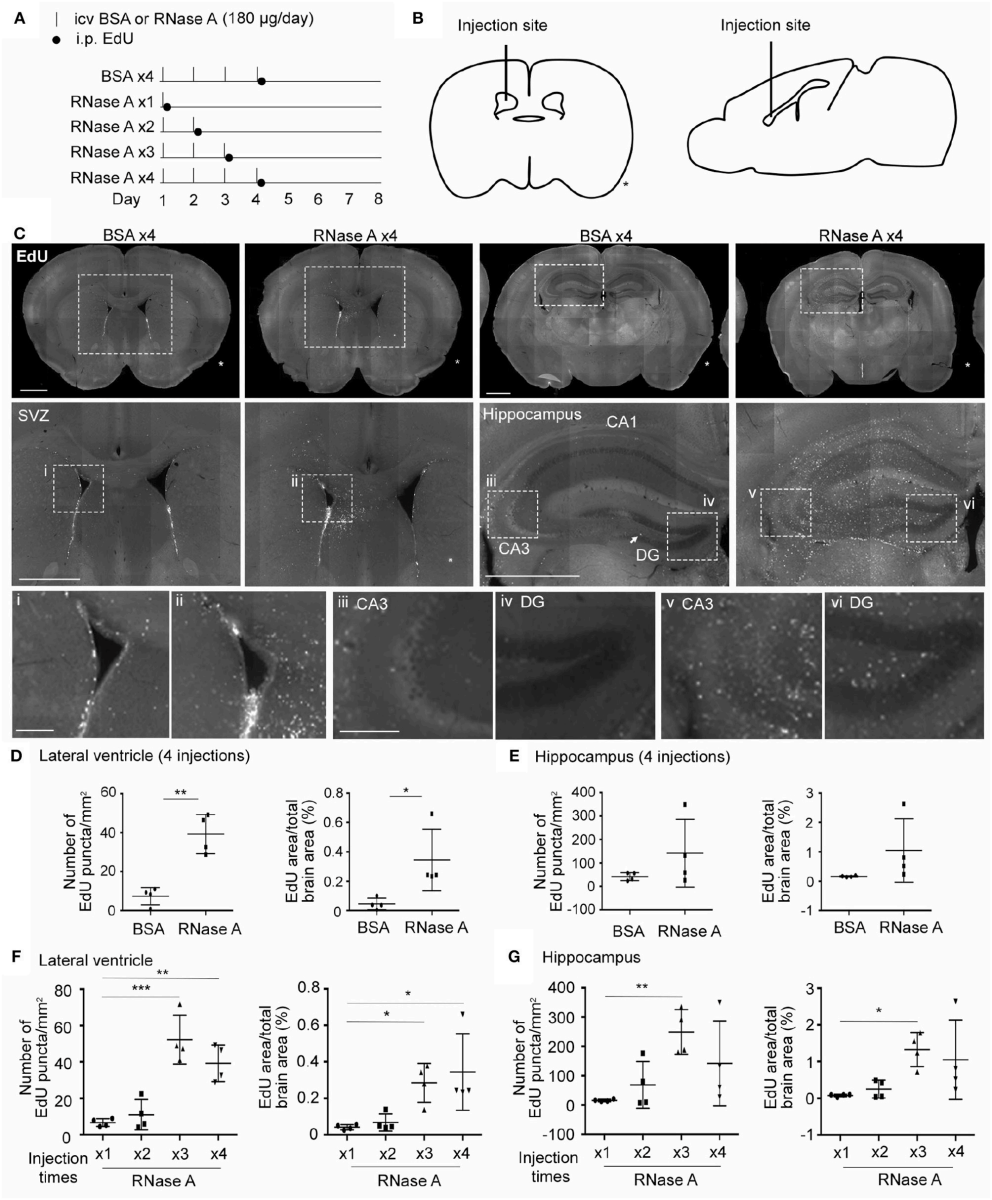
RNase A treatment induces EdU incorporation in mouse brains. **(A)** Schematic timeline for RNase A (Qiagen) treatment and EdU labeling. Intracerebroventricular (icv) injection of 180 μg RNase A or BSA control was performed once per day for one to four days, as indicated. After the last injection of each group, mice received a single intraperitoneal (i.p.) injection of EdU (100 mg/kg) to label proliferated cells. Mouse brains were harvested at day 8 after the first icv injection. **(B)** Schematic diagram showing the position of the icv injection. * indicates the non-injected side. **(C)** Representative images of EdU labeling of the BSA x4 and RNase A x4 groups in the subventricular region of the lateral ventricle (SVZ) and hippocampus. Images in the middle panel of **(C)** are enlargements of the squares in the respective upper panel; scale bar, 1 mm. Arrow points a EdU-positive cell at subgranular zone of dentate gyrus. Bottom panel of **(C)**; images (i, ii: SVZ; iii, v: zone CA3 of hippocampus; iv, vi: dentate gyrus, DG) are enlargements of the squares in the middle panels; scale bar, 200 μm. **(D–G)** Quantification of EdU-positive cells in both sides of the **(D, F)** lateral ventricle and **(E, G)** hippocampus. The same datasets of RNase A x4 are used in **(D, F)** and **(E, G)**. Data represent mean ± SD (*n* = 4 mice per group). *P < 0.05; **P < 0.01; ***P < 0.001. **(D, E)** Unpaired *t*-test or Mann-Whitney test; **(F, G)** One-way ANOVA.

We then investigated whether RNase A is able to promote NPC proliferation in mouse brains. RNase A was injected daily into one of the lateral ventricles of mouse brains for 1, 2, 3, or 4 days. After the last dose of RNase A, the thymidine nucleoside analog 5-ethynyl-2′-deoxyuridine (EdU) was intraperitoneally injected into mice to label dividing cells. Four doses of BSA (BSA x4) were performed as a control. Mice were then sacrificed to monitor EdU signals at day 8, i.e., 7 days after the first shot of RNase A or BSA had been injected (Figures 8A,B). Since RNase A was injected into lateral ventricle, we expected to find more EdU-positive cells at subventricular zone in RNase A treated mice. Indeed, compared with the BSA control (BSA x4), the four injections of RNase A (RNase A x4) noticeably increased EdU puncta in periventricular zone (Figure 8C). Two parameters were measured to quantify the effect of RNase A on neurogenesis. We first measured the total numbers of EdU^+^ nuclei. Since some EdU^+^ cells tended to aggregate, it was difficult to count individual cells. Therefore, as a second measure, we established the areas of EdU signal. Compared with BSA controls, both parameters indicated that the four doses of RNase A increased EdU labeling in mouse brains (Figures 8C–E). In terms of a dosage effect, either three or four doses (but not one or two) of RNase A noticeably increased EdU signals (Figures 8F,G). Thus, our results suggest that, similar to the results for neuronal cultures, intracerebroventricular injection of RNase A promotes NPC proliferation *in vivo*.

**Figure 9:**
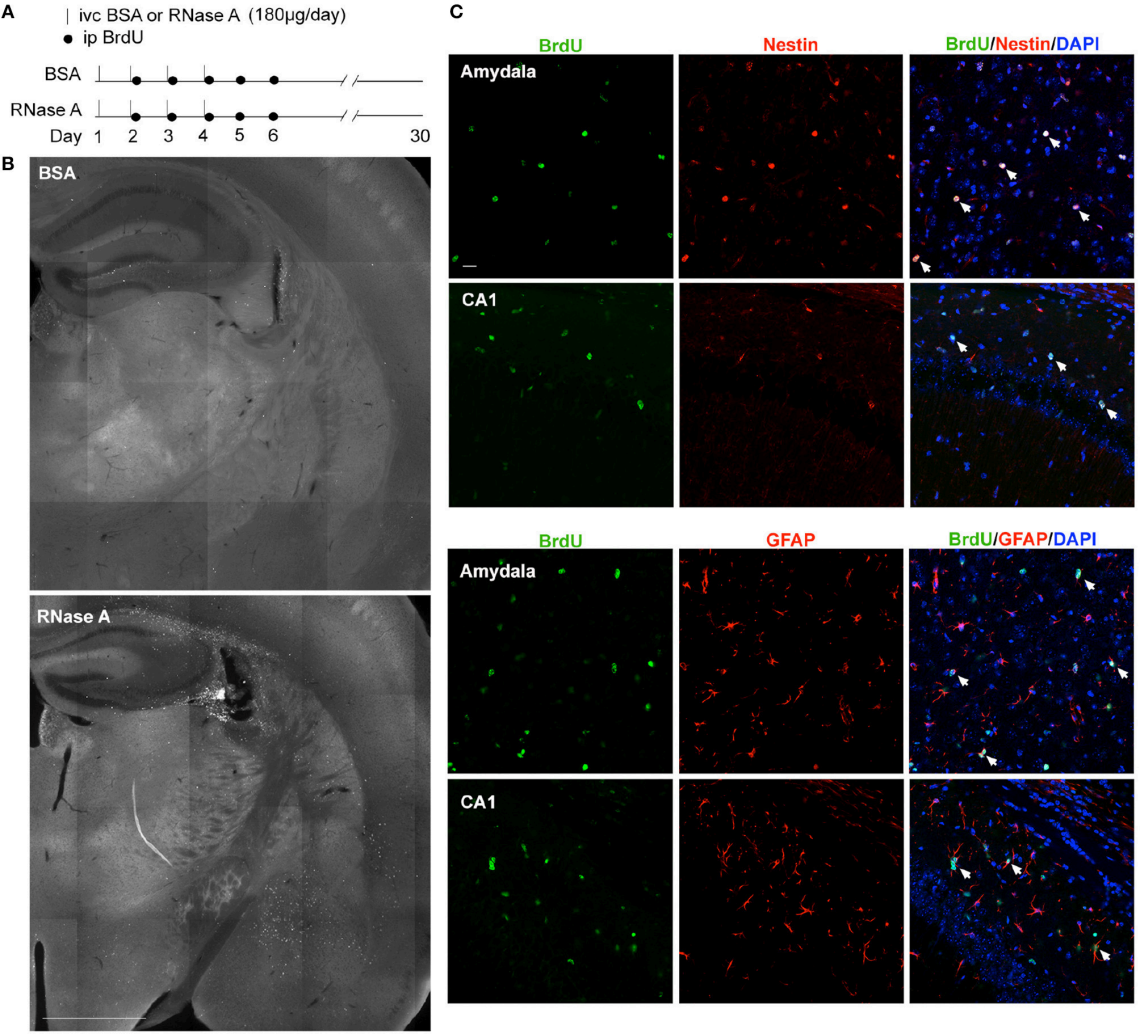
RNase A-induced NPCs migrate to various brain regions. **(A)** Schematic timeline for RNase A (Qiagen) injection into lateral ventricles and BrdU labeling *in vivo*. **(B)** BrdU staining 30 days after the first BSA or RNase A injection. Upper, BSA group; lower, RNase A group. **(C)** Double immunostaining with BrdU and Nestin or GFAP antibodies. Counter-staining with DAPI was performed. The results for the amygdala and hippocampal CA1 region are shown. Note that Nestin was concentrated at the nuclei of migrating NPCs. White arrows indicate some double-positive cells. Scale bars, **(B)** 1 mm; **(C)** 20 μm.

Although RNase A was injected into the lateral ventricle, we noticed that EdU-positive cells were not restricted to the subventricular zone. Instead, they were distributed over the entire hippocampus and in other brain regions (Figures 8C–G). It seems that the EdU-positive cells could migrate from the subventricular zone into other brain regions. To further assess this behavior, we modified our *in vivo* labeling experiments by injecting more BrdU and monitoring the distribution of BrdU-positive cells a month later (Figure 9A). In the BSA control group, we found very few BrdU-positive cells, and they were concentrated in the subgranular zone of the dendate gryrus and the region surrounding the lateral ventricle (Figure 9B, upper). In the RNase A group, we observed numerous BrdU-positive cells in the hippocampus, amygdala, cortex, thalamus, and striatum (Figure 9B). These BrdU-positive cells were Nestin- and GFAP-positive (Figure 9C), but Iba1-negative (Figure S4), suggesting that they are a lineage of NPCs but not microglial cells. We noticed that Nestin proteins were highly concentrated in the nuclei of BrdU-positive cells (Figure 9C), consistent with a recent report indicating that nuclear Nestin is involved in preventing cell senescence (Zhang et al., 2018). These data suggest that RNase A-induced NPCs are able to migrate to various brain regions and they further support the role of RNase A in promoting NPC proliferation and maintenance.

## Discussion

We unexpectedly found that RNase A can trigger NPC proliferation. RNase A purchased from either Invitrogen or Qiagen promotes BrdU incorporation and increases numbers of NPCs in dissociated neuronal cultures as well as neurosphere cell mass. Ara-C treatment assay further suggests that the effect of RNase A on NPCs is to enhance proliferation. Our study also indicates that the ERK pathway is critical for the effect of RNase A on NPC proliferation. Addition of U0126 to inhibit the ERK pathway efficiently neutralized the triggering effect of RNase A on NPC proliferation. Finally, we have also demonstrated that local infusion of RNase A into mouse cerebroventricles noticeably promotes EdU incorporation, reflecting entry of cells into S phase, *in vivo*. Thus, RNase A possesses an unexpected function of triggering NPC proliferation both *in vitro* and *in vivo*.

Studies have shown that cell surface receptors, including the transmembrane proteins heparan sulfate proteoglycan syndecan-4 (Skorupa et al., 2012), Plexin-B2 (Yu et al., 2017), and EGFR (Wang et al., 2018), are involved in the binding, internalization, and signaling of RNase A and angiogenin, another member of the RNase A superfamily (Chao and Raines, 2011; Gupta et al., 2013; Yu et al., 2017; Wang et al., 2018). EGFR is the common cell surface receptor of RNase A and angiogenin to trigger proliferation of pancreatic cancer cells (Wang et al., 2018). Bovine RNase A binds to EGFR and transmits EGFR downstream signaling, including ERK activation, in various types of cancer cells (Wang et al., 2012). Since the EGFR pathway is also well-known to promote neurogenesis (Mahanthappa and Schwarting, 1993; Ferri and Levitt, 1995; Wong and Guillaud, 2004), it is likely that the EGFR pathway is involved in the effect of RNase A on promoting NPC proliferation. ERK activation by RNase A treatment in neuronal cultures echoes this speculation. Moreover, the cancer cell study showed that the enzymatic activity of RNase A is not involved in the proliferation of cancer cells (Wang et al., 2018), so if RNase A uses the same mechanism to promote NPC proliferation, its enzymatic activity may not be critical for its effect. We found that ERK activity was increased 10 min after RNase A treatment. It is likely that RNase A binds to a EGFR and then activates an ERK pathway. In addition to activation of cell surface receptor, members of the RNase A superfamily are known to influence innate immune responses through RNA degradation and consequent regulation of RNA-binding TLRs or other nucleotide sensors in cells (Gupta et al., 2013; Liu et al., 2013; Chen et al., 2014). In particular, it has previously been suggested that TLR3 plays a role in neurogenesis (De Miranda et al., 2010; Okun et al., 2010; Forrest et al., 2012). Although TLR3 preferentially binds dsRNA, it can still recognize incomplete stem structures in viral ssRNA (Tatematsu et al., 2013). Thus, RNase A degradation of ssRNA might influence the activity of TLR3. Indeed, in a study of myocardial ischemia-reperfusion injury, RNase A was shown to reduce serum RNA levels and to attenuate TLR3 activation in the injury pathway (Chen et al., 2014). It would be interesting to explore further whether the innate immune receptors play a role in RNase A-mediated NPC proliferation. Enzymatic activity-dependent and -independent pathways do not mutually exclude from each other. It will be intriguing to explore the possibility in the future.

We used bovine RNase A to stimulate NPC proliferation. The bovine and human orthologs of RNase A (a rapidly evolving gene) share ~70% amino acid sequence identity. It is unclear whether RNase A from humans or other species also triggers NPC proliferation. Nevertheless, another two members of the RNase A superfamily, namely RNase 4 and angiogenin, also possess the ability to promote cell proliferation (Li et al., 2013; Yu et al., 2017). Apart from promoting cell proliferation, recent studies have indicated that members of the RNase A superfamily have cytotoxic and anti-tumor activities. *Rana catesbeiana* RNase A induces cell death of human glioblastoma cell lines (Chen et al., 2015). Intramuscular injection of RNase A suppresses growth and metastasis of transplanted murine Lewis lung carcinoma in mice (Mironova et al., 2013, 2017). Use of mutated human RNases as anti-tumor drugs has been suggested (Suzuki et al., 1999; Attery et al., 2018). However, our results and a recent study (Wang et al., 2018) evidence a role of bovine RNase A in promoting proliferation of NPCs and various cancer cells, suggesting that RNase A exhibits versatile activities in different cell types and also that different RNases have differing biological activities. Thus, caution should be exercised when applying RNases, particularly *in vivo*.

## Author contributions

H-YL designed and performed the majority of experiments, blind analyzed the data, and wrote the manuscript. C-YC designed and performed the experiments of Ara-C inhibition and ERK phosphorylation and wrote the manuscript. Y-FH, H-RL, and T-NH performed and assisted with the *in vivo* experiment. H-WC performed the time-lapse recording to monitor NPC proliferation and wrote the manuscript. W-PL contributed mock control experiment and wrote the manuscript. C-NC and P-YS assisted with biochemical analysis. Y-PH designed experiments, supervised the project, secured financial support, and wrote the manuscript. All authors read and approved the final version of manuscript.

### Conflict of interest statement

The authors declare that the research was conducted in the absence of any commercial or financial relationships that could be construed as a potential conflict of interest.
