# Supplementary material for: RNase A Promotes Proliferation of Neuronal Progenitor Cells via an ERK-Dependent Pathway
Source: Front Mol Neurosci. 2018 Nov 26;11:428. doi: 10.3389/fnmol.2018.00428 (PMC6275325; doi:10.3389/fnmol.2018.00428)
Supplement: Supplementary file 2 [file Data_Sheet_1.pdf]

Supplementary Information

RNase A promotes proliferation of neuronal progenitor cells via an ERK-dependent pathway

Hsin-Yu Liu, Chiung-Ya Chen, Yun-Fen Hung, Hong-Ru Lin, Hsu-Wen Chao, Pu-Yun Shih, Chi-Ning Chuang, Wei-Ping Li, Tzyy-Nan Huang and Yi-Ping Hsueh

Institute of Molecular Biology, Academia Sinica, Taipei, 11529, Taiwan

Context: 1 Supplementary Movie, 4 Supplementary Figures, 1 Supplementary Table

Movie S1. Dividing cells are present in neuronal culture (related to Figure 2B). Time-lapse recording of neuronal culture was conducted from DIV 0 to 4. Red arrowhead points to a cell aggregate containing NPCs, which were actively dividing during recording.

Figure S1. AKT is not activated by RNase A treatment.
Figure S2. Step-by-step elucidation of quantification of EdU$^+$ cell density in brain.
Figure S3. Step-by-step elucidation of quantification of EdU$^+$ cell density in hippocampus.
Figure S4. BrdU and Iba1 double-staining after *in vivo* administration of RNase A (related to Figure 9).
Table S1. Antibody information.

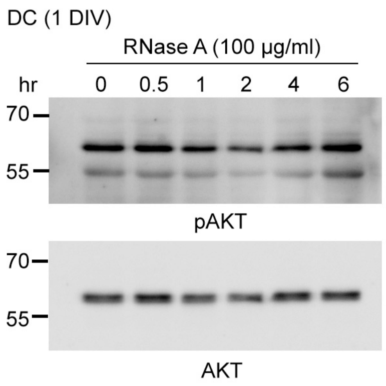

Supplementary Figure S1. AKT is not activated by RNase A treatment. Cortex and hippocampus dissociated cultures (DC) were treated with RNase A (100 μg/ml) at 1 DIV for different time periods, as indicated, and harvested for immunoblotting with phospho-AKT (Thr308) and pan-AKT antibodies. Unlike ERK (shown in Figure 8), no obvious AKT phosphorylation was found upon RNase A treatment.

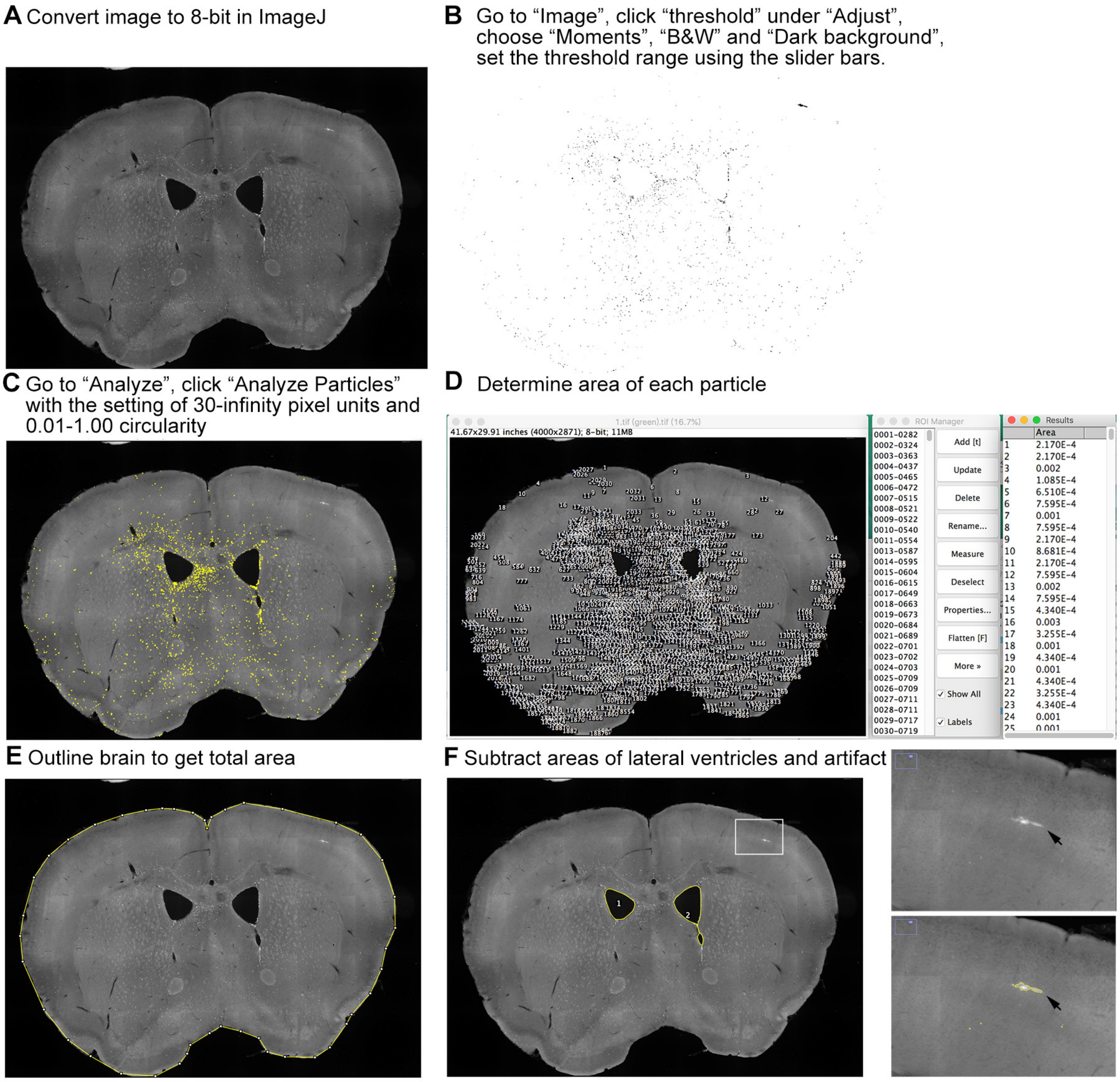

Supplementary Figure S2. Step-by-step elucidation of quantification of EdU^{+} cell density in brain. (**A**) Images were converted into 8-bit. (**B**) Images were adjusted with a threshold (“Moments” and “B&W” modes in “Dark background”) using ImageJ software. (**C**) The area of fluorescence reactivity was analyzed using “Analyze Particles” with the setting of 30-infinity pixel units and 0.01-1.00 circularity. (**D**) The area of each particle was determined. (**E**) The brain was outlined to get total area. (**F**) Areas of lateral ventricles and artifacts (arrow as an example) were further subtracted.

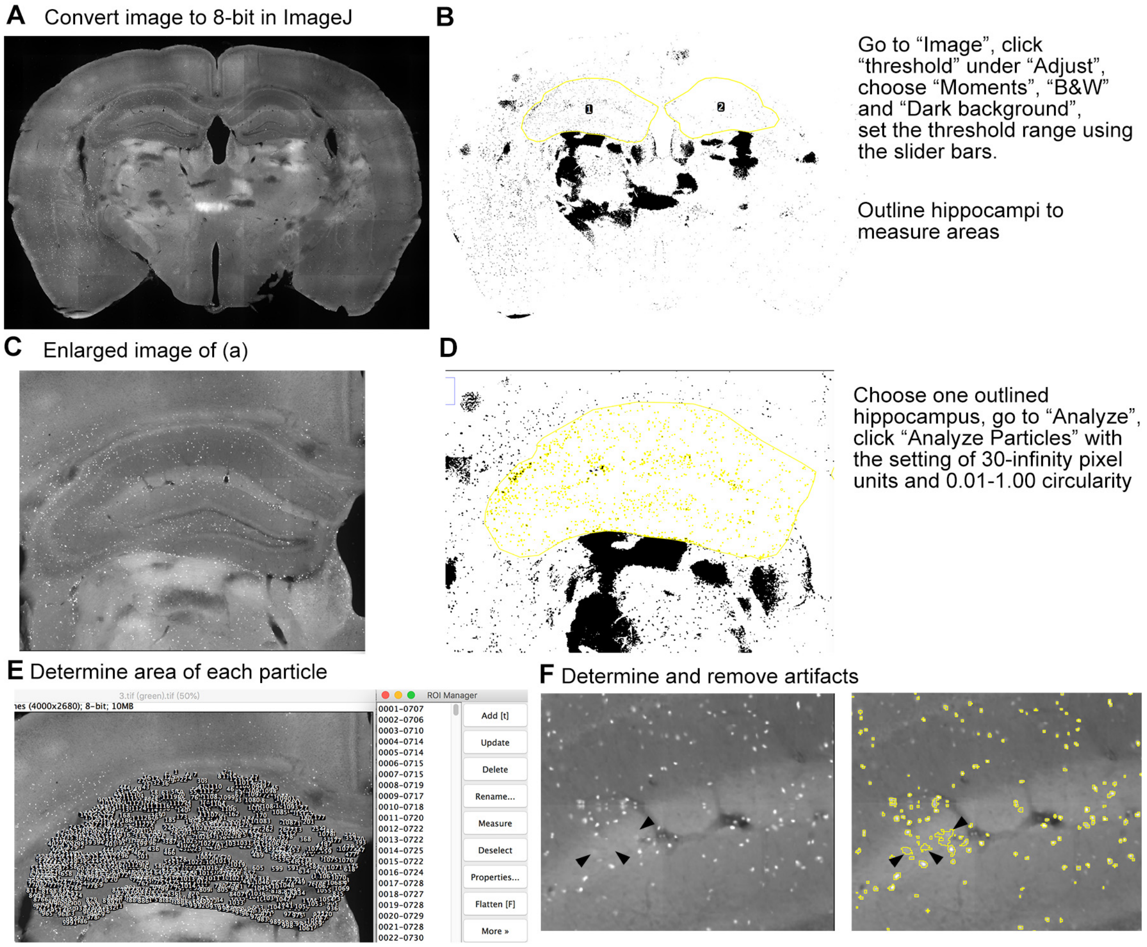

Supplementary Figure S3. Step-by-step elucidation of quantification of EdU$^+$ cell density in hippocampus. (**A**) Images were converted into 8-bit. (**B**) Images were adjusted with a threshold (“Moments” and “B&W” modes in “Dark background”) using ImageJ software. Outline hippocampus to get areas. (**C**) Enlarged image of left side of hippocampus. (**D**) The area of fluorescence reactivity was analyzed using “Analyze Particles” with the setting of 30-infinity pixel units and 0.01-1.00 circularity. (**E**) The number and area of each particle was determined. (**F**) The quantitative results were manually corrected to remove artifacts (arrowheads as examples).

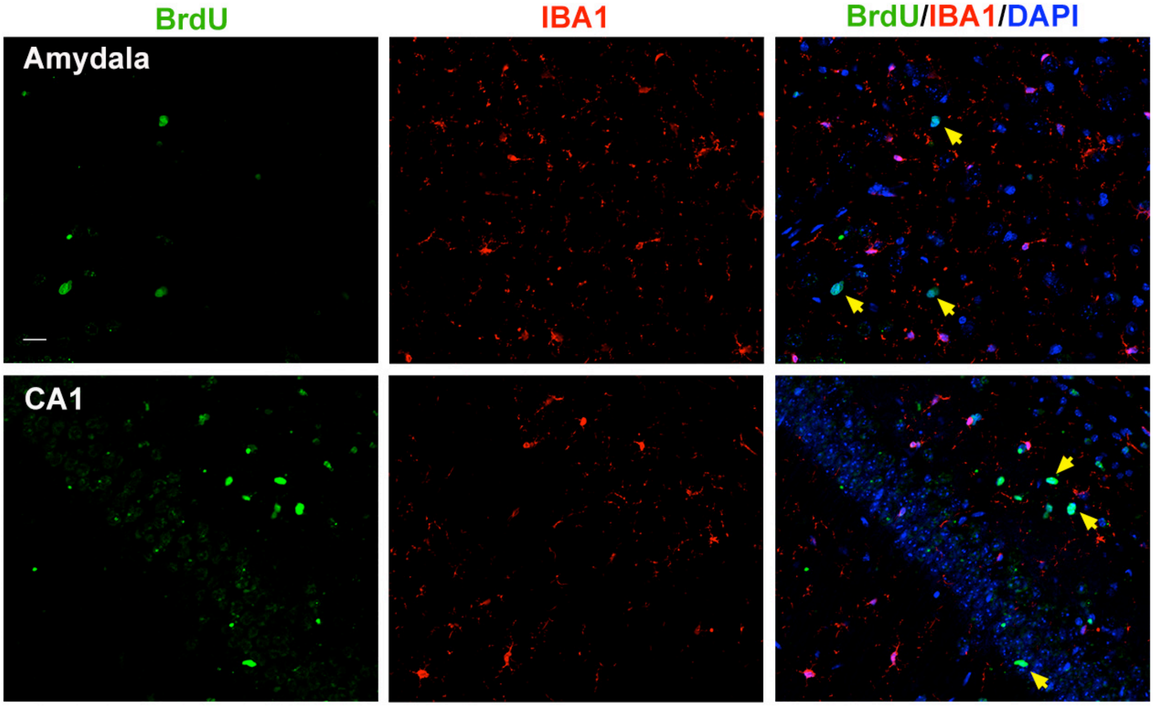

Supplementary Figure S4. BrdU and Iba1 double-staining after *in vivo* administration of RNase A (related to Figure 9). Iba1-positive microglial cells were not labeled by BrdU after RNase A treatment *in vivo*. Yellow arrows indicate some BrdU-single-positive cells. Scale bar, 20 µm.

Supplementary Table 1. Summary of primary antibodies

Antigen	Host species	Source	Working concentration
MAP2	Rabbit	Millipore	ICC 1:500
Nestin	Mouse	Millipore, MAB353	ICC 1:500
			IHC 1:250
BrdU	Rat	Abcam, ab6326	ICC 1:500
			IHC 1:200
SOX2	Rabbit	Abcam, ab97959	ICC 1:500
GFAP	Mouse	Chemicon, MAB3402	ICC 1:500 (1 μg/ml)
IBA1	Rabbit	Wako, 019-19741	ICC 1:500 (0.5 μg/ml)
p-ERK1/2	Rabbit	Cell signaling (20G11) (Thr202/tyr204), #4376	WB 1:1000
ERK (pan)	Rabbit	Cell signaling, #4695	WB 1:1000
p-AKT (Thr308)	Rabbit	Cell signaling, #9275	WB 1:1000
AKT (pan)	Rabbit	Cell signaling, #4691S	WB 1:1000
VCP	Mouse	BD, 612183	WB 1:1000
